# The Spectrum of Histopathological Lesions in Children Presenting with Steroid-Resistant Nephrotic Syndrome at a Single Center in Pakistan

**DOI:** 10.1100/2012/681802

**Published:** 2012-05-02

**Authors:** Muhammed Mubarak, Javed I. Kazi, Shaheera Shakeel, Ali Lanewala, Seema Hashmi

**Affiliations:** ^1^Histopathology Department, Sindh Institute of Urology and Transplantation (SIUT), Karachi 74200, Pakistan; ^2^Pediatric Nephrology Department, Sindh Institute of Urology and Transplantation (SIUT), Karachi 74200, Pakistan

## Abstract

Steroid-resistant nephrotic syndrome (SRNS) is a common problem in pediatric nephrology practice. There is currently little information in the literature on the spectrum of histopathologic lesions in children presenting with SRNS in Pakistan. This study was designed to determine the histopathologic lesions in children presenting with SRNS at our center. The study was conducted at the Histopathology Department, Sindh Institute of Urology and Transplantation (SIUT) from January 2009 to August 2011. All children (≤16 years) presenting with SRNS, in whom renal biopsies were performed, were included. Their demographic, clinical, laboratory, and histopathological data were retrieved from files and original renal biopsy forms. The results were analyzed by SPSS version 10.0. A total of 147 children were included. Of these, 91 (61.9%) were males and 56 (38.1%) females, with male-to-female ratio of 1.6 : 1. The mean age was 7.03 ± 4.0 years (range: 6 months–16 years). The histopathological lesions seen on renal biopsies comprised of focal segmental glomerulosclerosis (FSGS) (38.5%), followed by minimal change disease (MCD) (23.2%), IgM nephropathy (IgMN) (13.6%), idiopathic mesangial proliferative GN (10.2%), membranous GN (8.2%), and mesangiocapillary GN (4.8%). Our results indicate that FSGS is the predominant lesion in children with SRNS, followed by MCD and IgMN.

## 1. Introduction

Steroid-resistant nephrotic syndrome (SRNS) is a common problem in pediatric nephrology practice and one that poses significant therapeutic challenge for pediatric nephrologists [1–4]. The children with SRNS tend to progress to end-stage renal disease (ESRD) due to the progressive damage of the glomerular filtration barrier (GFB) [5–8]. The frequency of SRNS among all cases of idiopathic nephrotic syndrome (INS) varies throughout the world [9–13]. Reported rates of steroid resistance among the biopsy series vary from 10 to 20% in different studies. We have found earlier a steroid-resistant pattern of INS in 30% of our children who underwent renal biopsy [14]. There are very few studies in the literature on the histopathological spectrum of glomerulopathies underlying SRNS in children [9–12]. Some authors have suggested that the underlying histopathology affects the course of SRNS and its response to treatment. There is currently little information about the histopathologic spectrum of lesions in children presenting with SRNS in Pakistan [15, 16]. There are also data in the literature that suggest that the pattern of histopathology underlying INS is changing not only in adults but also in children [17–20]. It then became imperative to determine the true pattern of the prevailing glomerulopathies underlying SRNS in a large cohort of children who presented to our center.

This study was thus designed to determine the spectrum of histopathological lesions in children presenting with SRNS at our center.

## 2. Material and Methods

This descriptive, observational study was conducted from January 2009 till August 2011 at the Department of Histopathology, Sindh Institute of Urology and Transplantation (SIUT). All children presenting with SRNS at the Pediatric Nephrology Outpatient Department of SIUT and in whom renal biopsies were performed during the above-mentioned period were included. Their demographic, clinical, and laboratory data at the time of presentation and on last followup were retrieved from case files. The histopathological findings including light microscopy (LM) and immunoflourescence (IF) findings were recorded from original renal biopsy forms. Standard definitions of the disease and treatment responses were used as in our previous study [14]. Informed consent was obtained from the children or their parents before obtaining real-time ultrasound-guided percutaneous renal biopsies by automated biopsy gun.

### 2.1. Pathologic Study

At our center, two cores of native renal biopsy are routinely obtained for full pathologic evaluation as described in detail in our previous paper [14]. One core is processed for LM and fixed in 10% buffered formalin. The other core is divided into two pieces under dissection microscope for electron microscopic (EM) and IF study. All biopsies were examined by the same two experienced renal pathologists (J. I. Kazi and M. Mubarak) jointly in close liaison with nephrologists to arrive at the best possible correlative diagnosis. This ensured consistency and uniformity of diagnostic categories and the pathological diagnoses throughout the study period.

### 2.2. LM

For LM, routinely 10 serial sections are cut and stained by hematoxylin and eosin (H and E), Masson's trichrome, periodic acid-Schiff (PAS), and silver stains (Gomori's methenamine silver, GMS). In our laboratory, renal tissue sections are cut at a thickness of 2 um for optimal evaluation of the morphologic details as reported previously [14].

### 2.3. IF

Tissue specimens for IF are snap-frozen in liquid nitrogen and cut on cryotome. The tissue is stained by the direct method using fluorescein isothiocyanate- (FITC-) conjugated antisera monospecific for IgG, IgA, IgM, C3, and C1q (Dako, Glostrup, Denmark). The slides are visualized under the epifluorescense microscope and graded semiquantitatively as 0 to 3+ (on a scale of 0 to 3+, where 0 = absent and 3+ = brightest) and distribution described as membranous or mesangial in a granular or liner pattern as described previously [14]. IF findings on the biopsy specimens were obtained from the original renal biopsy reports.

### 2.4. EM

Tissue samples for EM were processed as described in our previous report [14]. Briefly, EM tissue was fixed in 4% glutaraldehyde, postfixed in 1% osmium tetroxide at 0.02 M Sorenson Phosphate buffer at pH 7.4, processed for EM, and embedded in Eponate resin. Ultra-thin sections (100 nm) were cut on Leica ultramicrotome. Sections were stained on copper 300-mesh girds with Uranyl acetate and Lead citrate and examined with a JEM 1200 EX II electron microscope.

### 2.5. Final Diagnosis

**Figure 1:**
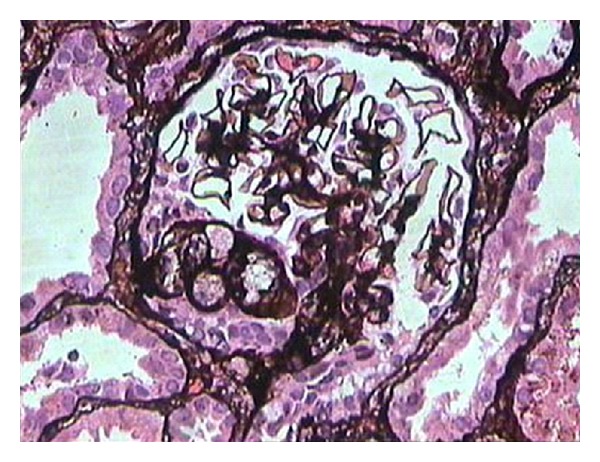
Medium-power view showing a glomerulus with segmental scarring associated with intracapillary foam cells and adhesion formation with Bowman's capsule, in a case of classic focal segmental glomerulosclerosis. (Silver stain, ×200).

**Figure 2:**
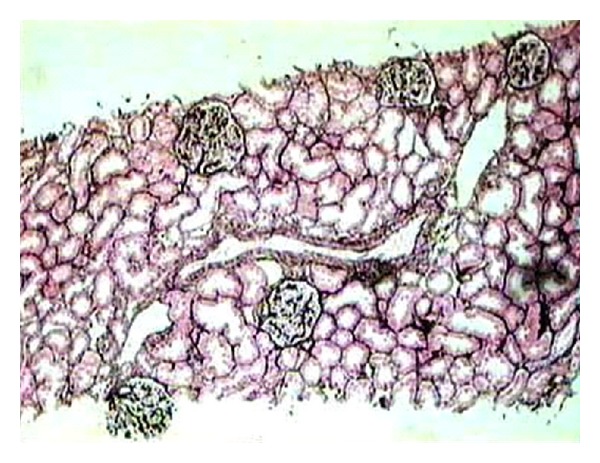
Low-power view of a representative section of a renal biopsy showing five glomeruli with minor changes. There is no segmental scarring or significant mesangial proliferation. One small artery included as well as the surrounding tubulointerstitial compartment show no significant pathology. This case was diagnosed as minimal change disease after IF study showed negative results and EM showed diffuse effacement of foot processes. (Silver stain, ×100).

**Figure 3:**
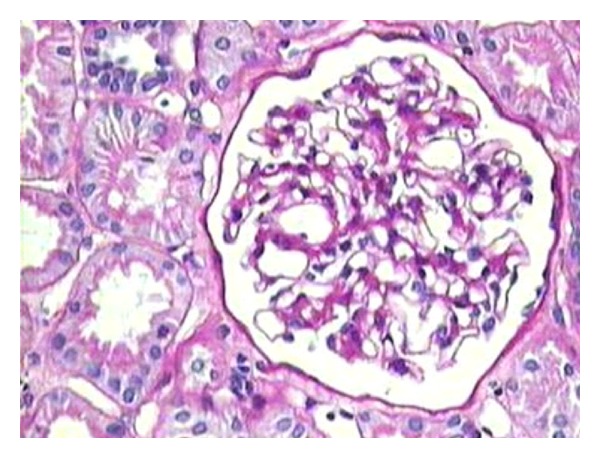
Medium-power view showing a glomerulus with mild mesangial prominence, but no significant increase in mesangial hypercellularity. This case latter turned out to be IgM nephropathy after IF showed diffuse granular mesangial positivity of IgM, as shown in Figure 4. (PAS stain, ×200).

**Figure 4:**
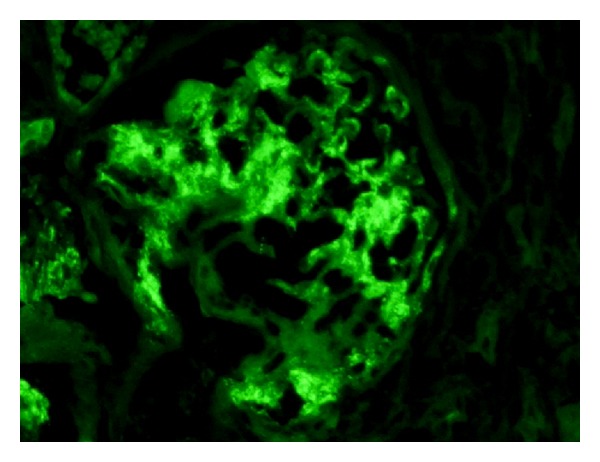
Medium-power view showing a glomerulus with diffuse granular mesangial positivity of IgM on IF study. (IgM, ×200).

This was made by correlating the entire clinical, laboratory, and the pathological findings. The final diagnosis was retrieved from the original renal biopsy reports. Standard definitions of the diseases were utilized for the pathological categorization of the glomerular lesions as in our previous study, and shown in Figures 1–4 [14].

### 2.6. Statistical Methods

Statistical analysis was carried out using IBM compatible SPSS for windows version 10.0 (SPSS, Chicago, IL, USA). Simple descriptive statistics such as mean ± SD were used for variables such as age and clinical and laboratory features. Percentages were used for categorical data.

## 3. Results

A total of 147 pediatric patients with SRNS and in whom percutaneous renal biopsies were performed were included. Of these, 91 (61.9%) were males and 56 (38.1%) females with the male-to-female ratio of 1.6 : 1. The mean age at presentation was 7.03 ± 4.0 years with a range of 6 months to 16 years. Majority of children were young, with only 21% belonging to the adolescent age group (12–16 years). The demographic characteristics of all children with SRNS are given in Table 1.

The spectrum of histopathological lesions seen on renal biopsies was wide and comprised of focal segmental glomerulosclerosis (FSGS) (38.7%), followed by minimal change disease (MCD) (23.1%), IgM nephropathy (13.6%), idiopathic mesangial proliferative GN (MesPGN) (10.2%), membranous GN (MN) (8.2%), mesangiocapillary or membranoproliferative GN (MPGN) (4.8%), and a number of rare lesions (Table 2). These results demonstrate the predominance of FSGS in children with SRNS. MCD was second in frequency, followed by IgMN. The later disease is a relatively recently described and still largely controversial entity, which is again at the forefront of nephropathology literature. A comparison of the common histopathological lesions in our study with local and international studies is given in Table 3.

## 4. Discussion

This is one of the largest studies on the spectrum of histopathological lesions underlying SRNS in children and adolescents from a single center located in the southern metropolitan city of Karachi in Pakistan. We believe that the results of this study represent the true pattern of glomerulopathies in children presenting with SRNS from this part of the world. This is because our renal pathology laboratory is the only laboratory in the country that is equipped with all the necessary modalities including IF and EM that are required for the complete evaluation of renal biopsies. This may seem a routine matter in most of the laboratories in the developed world, but the situation in developing countries is just the opposite. Moreover, although our center is located in the southern part of the country and our catchment area mostly comprises of southern provinces of Sindh and Balochistan, but we receive patients from all over the country. This is because SIUT offers free consultation and treatment to all patients with kidney diseases [21].

There is still controversy over the role of renal biopsy in the management of SRNS children [12]. Earlier reports suggested that the outcome of the disease can be predicted from the clinical response to steroids, and the biopsy is unnecessary for a vast preponderance of children with INS [9]. More recent evidence suggests that the histopathological spectrum of glomerulopathies underlying INS is changing in both adults and children [17–20]. Many authors have found a rise in the prevalence of FSGS in children during the recent past, and since this lesion is associated with a significantly lower response to steroids than MCD, it is recommended to detect its presence on renal biopsy in order to better inform the patients about the long-term prognosis. These authors also recommend a renal biopsy before potentially nephrotoxic drugs, such as Cyclosporine are started [7, 12, 14, 16].

The results of our study are generally similar to those previously reported in the literature. All the previously reported studies have observed a higher prevalence of FSGS in this form of INS [10–13]. The incidence of this lesion is increasing throughout the world not only in adults but also the children [7]. The rates of FSGS diagnosis are not uniform across the world, however. A study from India found FSGS in 50% of cases of SRNS children [12]. A similar rate was also reported in the studies from Saudi Arabia and Tunisia [13, 22]. In contrast, FSGS was less common in studies from Japan, France, and Kuwait [3, 6, 8]. The reasons for the discrepancies in the results are not exactly known, but may be related to racial, genetic, or environmental factors. Moreover, slight differences in disease definitions and inclusion criteria may be partly responsible. One important factor, which should also be considered, is that of observer variation in the reporting of renal lesions, especially of MCD, mesangial proliferative GN, and early FSGS [14, 22]. The lesions of early FSGS may be missed easily if not sought carefully by examining multiple sections of the renal biopsy. All our biopsies were examined by trained renal pathologists with vast work and research experience, and it is extremely unlikely that we missed the diagnosis of this lesion. The frequency of FSGS was slightly less than that observed in the subgroup of INS with SRNS in our earlier report [14]. The exact reason for this change is not known but may be partly due to the lower threshold of upper age limit of children included in the present study. It is worthy to mention here that the adolescents constituted only 21% of all children with SRNS in the present study.

Overall, MCD is the most common cause of INS in children, especially under six years of age. However, its incidence in SRNS is lower than that of FSGS in most of the reported studies [10–13]. Our findings also concur with these studies. Only a few studies found MCD as more common than FSGS in children with SRNS [3, 6, 8]. The reasons for this paradoxical finding are not known but may be related to environmental, genetic, or racial factors [22].

IgMN is a relatively new entrant to the list of glomerulopathies underlying INS both in children and adults. However, its status as a distinct entity or even its existence is under debate till date. The disease was first described in 1978 by two independent groups of investigators [23, 24]. A flurry of publications soon followed from different parts of the world, but the interest in the disease soon faded in the western world [25]. More recently, a number of reports have emerged mostly from tropical countries [26, 27]. We have also published our experience of the disease in one of the largest studies of IgMN in children presenting with INS at our center [21]. In the current study, IgMN was found in 13.6% of children with SRNS. There is a need of collaboration between the nephrology centers in the developed and the developing countries for undertaking basic research to explore the etiology and the pathogenesis of the condition.

MN was less common in our children with SRNS as in almost all previously published series on this subject [10–16].

In conclusion, our results indicate that FSGS is the predominant lesion in children with SRNS, followed by MCD and IgMN. The study defines the true spectrum of histopathological lesions underlying SRNS in children in Pakistan.

## Figures and Tables

**Table 1:** Patient characteristics.

Total number of patients	147
Males	91 (61.9%)
Females	56 (38.1%)
Male-to-female ratio	1.6 : 1
Mean age (in years)	7.03 ± 4.0
Age range	(6 months–16 years)

**Table 2:** The frequency distribution of different histopathological lesions in 147 pediatric patients with steroid-resistant nephrotic syndrome.

Pathological lesions	Frequency	Percentage
Focal segmental glomerulosclerosis	57	38.7
Minimal change disease	34	23.1
IgM nephropathy	20	13.6
Mesangioproliferative GN	15	10.2
Membranous GN	12	8.2
Mesangiocapillary GN	7	4.8
IgA nephropathy	1	0.6
Chronic sclerosing GN	1	0.6

Total	147	100

**Table 3:** Comparison of histopathological lesions of our study with local and international studies (all figures are in percentages).

Histopathological lesions	Our study	Olowu et al. [10]	Kari et al. [11]	Gulati et al. [12]	Azhar et al. [16]
FSGS	38.7	39.1%	39	58.8	28.8
MCD	23.1	4.3	8	17.6	13.3
IgMN	13.6	—	28	—	17.6
MesPGN	10.2	8.7	17	17.6	8.8
MN	8.2	4.3	—	1.4	13.3
MPGN	4.8	43.5	—	1.6	11.5
IgAN	0.6	—	3	—	6.6

FSGS: focal segmental glomerulosclerosis; IgANL: IgA nephropathy; IgMN: IgM nephropathy; MCD: minimal change disease; MesPGN: mesangioproliferative GN; MN: membranous GN; MPGN: mesangiocapillary or membranoproliferative GN.
